# Author Correction: Machine learning-based model for prediction of clinical deterioration in hospitalized patients by COVID 19

**DOI:** 10.1038/s41598-022-12247-9

**Published:** 2022-05-12

**Authors:** Susana Garcia-Gutiérrez, Cristobal Esteban-Aizpiri, Iratxe Lafuente, Irantzu Barrio, Raul Quiros, Jose Maria Quintana, Ane Uranga, COVID-REDISSEC Working Group, Susana García-Gutiérrez, Iratxe Lafuente, Jose María Quintana, Miren Orive, Nerea Gonzalez, Ane Anton, Ane Villanueva, Cristina Muñoz, Maria Jose Legarreta, Raul Quirós, Pedro Pablo España Yandiola, Mikel Egurrola, Amaia Aramburu, Amaia Artaraz, Leire Chasco, Olaia Bronte, Patricia García, Ana Jodar, Virginia Fernandez, Cristobal Esteban, Naia Mas, Esther Pulido, Itxaso Bengoetxea, Antonio Escobar Martínez, Amaia Bilbao, Iñigo Gorostiza, Iñaki Arriaga, José Joaquín Portu Zapiarain, Naiara Parraza, Milagros Iriberri, Rafael Zalacain, Luis Alberto Ruiz, Leyre Serrano, Adriana Couto, Oier Ateka, Arantza Cano, Maria Olatz Ibarra, Eduardo Millan, Mayte Bacigalupe, Jon Letona, Andoni Arcelay, Iñaki Berraondo, Xavier Castells, Margarita Posso, Lilisbeth Perestelo, Guillermo Perez Acosta, Candelaria Martín Gonzñalez, Maximino Redondo, Maria Padilla, Adolfo Muñoz, Ricardo Saenz de Madariaga

**Affiliations:** 1 Osakidetza Basque Health Service, Research Unit, Galdakao-Usansolo University Hospital, Barrio Labeaga S/N, 48960 Galdakao, Vizcaya Spain; 2 Cambrian Intelligence SLU, Madrid, Spain; 3 Department of Applied Mathematics and Operational Research, University of the Basque Country, Leioa, Spain; 4 Internal Medicine Service, Hospital Costa del Sol, Malaga, Spain; 5 Osakidetza Basque Health Service, Respiratory Service, Galdakao-Usansolo University Hospital, Galdakao, Spain; 6 Kronikgune Institute for Health Services Research, Barakaldo, Spain; 7 Red de Investigación en Servicios Sanitarios y Enfermedades Crónicas (REDISSEC), Zaragoza, Spain; 8 Respiratory Group, Biocruces Bizkaia Health Research Institute, Barakaldo, Spain; 9 Intensive Care Unit, Galdakao-Usansolo Universitary Hospital, Galdakao, Spain; 10 Emergency Department, Galdakao-Usansolo Universitary Hospital, Galdakao, Spain; 11 Hospital at Home, Galdakao-Usansolo Universitary Hospital, Galdakao, Spain; 12 Research Unit, Basurto Universitary Hospital, Bilbao, Spain; 13 Respiratoty Service, Basurto Universitary Hospital, Bilbao, Spain; 14 Internal Medicine, Bioaraba Institute, Vitoria, Spain; 15 Research Unit, Bioaraba Institute, Vitoria, Spain; 16 Internal Medicine, Donostia Universitary Hospital, San Sebastián, Spain; 17 Respiratory Service, Santa Marina Hospital, Bilbao, Spain; 18 Pharmacy Service, Urduliz Hospital, Urduliz, Spain; 19 Central Services Osakidetza, Vitoria, Spain; 20 Department of Health, Basque Government, Vitoria, Spain; 21 Unidad de Calidad e Investigacion, Hospital del Mar, Barcelona, Spain; 22 Servicio de Evaluación y Planificación del Servicio Canario de la Salud, Canary Islands, Tenerife, Spain; 23 Intensive Care Unit, Complejo Hospitalario Universitario Insular-Materno Infantil de Gran Canaria, Gran Canaria, Spain; 24 Internal Medicine, Hospital Universitario de Canarias, Tenerife, Spain; 25 Research Unit, Hospital Costa del Sol, Marbella, Spain; 26 Unidad de Salud Digital ISCIII, Madrid, Spain

Correction to: *Scientific Reports* 10.1038/s41598-022-09771-z, published online 02 May 2022

The original version of this Article contained an error in Affiliation 2, which was incorrectly given as ‘Cambrian Intelligence, London, UK’. The correct affiliation is listed below:

Cambrian Intelligence SLU, Madrid, Spain

The original Article has been corrected.

